# Characterization of hepatitis B virus with complex structural variations

**DOI:** 10.1186/s12866-018-1350-1

**Published:** 2018-12-03

**Authors:** Kei Fujiwara, Kentaro Matsuura, Kayoko Matsunami, Etsuko Iio, Shunsuke Nojiri

**Affiliations:** Department of Gastroenterology and Metabolism, Nagoya City University Graduate School of Medical Sciences, 1 Kawasumi, Mizuho, Nagoya, Aichi 467-8601 Japan

**Keywords:** Hepatitis B virus, Genetic rearrangement, Complex structural variation, Hepatocyte nuclear factor 1 binding site

## Abstract

**Background:**

Hepatitis B virus (HBV) infection is one of the most serious public health issues. Recent HBV genetic research has revealed novel genetic rearrangements termed complex structural variations (SVs), which are composed of combinations of SVs such as insertions, deletions, and duplications. An extensive search was made for complex SVs of HBV and their characteristics were analyzed.

**Results:**

Fifty-five HBV strains with complex SVs were identified by analyzing genetic sequences of HBV with bioinformatical tools. Along with 15 HBV strains with complex SVs in a previous report, a total of 70 HBV strains harboring complex SVs were analyzed. Complex SVs in the HBV genome were located frequently between nt 1500 and 2000. Insertions were observed in 65/70 (92.9%) of HBV strains with complex SVs. As insertional motif sequences, hepatocyte nuclear factor 1 binding site, a sequence complementary to part of box α in enhancer II, and insertions of unknown origins were observed. The complex SVs were classified into six groups, and combination of insertion and deletion was observed more frequently than other patterns.

**Conclusion:**

Through an extensive search of HBV sequences, new strains with complex SVs were identified in this study. Characteristics of HBV with complex SVs were clarified by the analysis of 70 HBV strains harboring complex SVs. Further investigation is required to elucidate its role in pathogenesis of HBV-related liver disease.

**Electronic supplementary material:**

The online version of this article (10.1186/s12866-018-1350-1) contains supplementary material, which is available to authorized users.

## Background

Hepatitis B virus (HBV) infection causes acute hepatitis and chronic liver disease. Approximately 257 million people are estimated to be infected with HBV, as diagnosed by hepatitis B surface antigen (HBs-Ag) positivity, and HBV-related liver cirrhosis and hepatocellular carcinoma cause approximately 900,000 deaths annually [1]. With recent advances in anti-HBV drugs, treatment with nucleos(t)ide analogues (NA) to suppress HBV DNA is highly effective. However, treatment to cure the infection has not been achieved and patients with chronic hepatitis B require long-term treatment [2]. Therefore, treatment to achieve a cure in a short period of time is required. For this purpose, fundamental research leading to further understanding of viral replication and the viral genome structure is very important.

Human HBV is a member of the family Hepadnaviridae, the viral particle is composed of an enveloped nucleocapsid that contains partially double-stranded DNA of approximately 3200 base pairs (bps). The virus is classified into various genotypes, according to nucleotide differences of more than 8% [3] and, currently, 10 genotypes (A-J) have been reported [4]. HBV genotypes show distinct geographical distributions worldwide [5–7] and unique, genotype-related features. For example, HBV genotype G (HBV/G) is frequently observed in patients with HIV infection and HBV/G co-infects with other HBV genotypes [8]. HBV/D has a specific genetic sequence in the pre-S1 region that differs from other genotypes and, in that area, HBV/D shares the genetic pattern of HBVs from non-human primates [9]. Clinically, it has been reported that HBV genotypes are associated with the severity of liver disease or treatment response [10, 11]. The HBV genome features virologically and clinically important mutations and structural variations (SVs). Core promoter mutations A1762T/G1764A are related to viral transcription and hepatocarcinogenesis [12–14]. Deletions in the pre-S regions also have been reported to be correlated with hepatocellular carcinoma [15, 16]. In addition, a mutation of box α in the core upstream of regulatory sequence (CURS)/enhancer II, C1653T, has been reported to be correlated with hepatocellular carcinoma in patients with HBV/C [17, 18]. Intergenotypic recombinations have been analyzed by many researchers [19–21], and these sometimes are found as polymorphic genetic differences which modify genotypes. For example, strains of HBV/ B in Japan differ from those in China and Taiwan; HBV/B in China and Taiwan having recombined with HBV/C whilst HBV/B in Japan has not [10].

Generally, genomic SVs include canonical forms such as insertions, deletions, and duplications. However, recent studies have reported non-canonical forms of SVs as complex SVs [22–25]. Complex SVs are determined by multiple breakpoints whose origin cannot be explained by a single end-joining or DNA exchange event [23]; in other words, they are determined by the presence of two or more SVs at the same locus [24]. There is a growing awareness of complex SVs, but they are often partially or completely missed because of the insufficient technology to capture the entire complexity of the SVs [26]. Starting from the case report of an unusual HBV strain, with a combination of insertion, deletion, and duplication [27], complex SVs of HBV were sought and more than 10 HBV strains with complex SVs were discovered and architectures of the genetic rearrangement were analyzed in the first report of complex SVs in the HBV genome [9].

By continuing this extensive search of complex SVs of HBV, more strains with complex SVs were investigated, and the characteristics of complex SVs were further analyzed in this study.

## Results

### PubMed search of HBV strains with complex SVs

A search in PubMed for additional HBV strain with complex SVs retrieved 203 abstracts or full text articles, which were manually reviewed. However, none of these articles reported HBV strains with complex SVs.

### BLAST search for insertional motif sequences

**Fig. 1:**
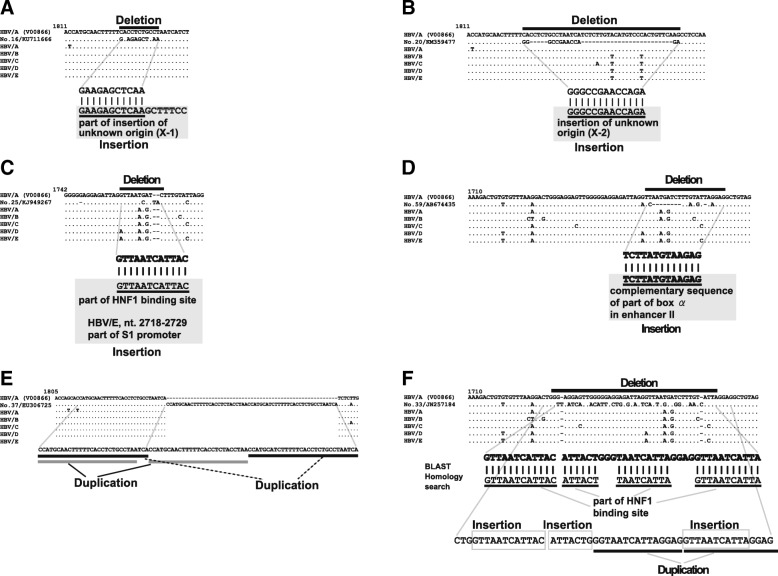
HBV strains with complex SVs. Some HBV strains with complex SVs analyzed in this study are shown. Other HBV strains with complex SVs analyzed in this study are shown in Additional file 3: Figure S1A to AM. A nucleotide alignment of the reference sequence of V00866 and consensus genetic sequences of HBV/A to HBV/E is shown. **a** Complex SV pattern of strain No. 16, composed of insertion of unknown origin (X-1) and deletion. **b** Complex SV pattern of strain No. 20, consisting of insertion of unknown origin (X-2) and deletion. **c** Complex SV pattern of strain No. 25, composed of HNF1 binding site insertion and deletion. **d** Complex SV pattern of strain No. 59, consisting of insertion of a sequence complementary to part of box α in enhancer II and deletion. **e** Complex SV pattern of strain No. 37, composing of two duplications. **f** Complex SV pattern of strain No. 33, consisting of three insertions of partial HNF1 binding sites and duplication, categorized as class VI, highly complicated (four or more SVs) in the classification in Table 2

Fifteen HBV strains with complex SVs reported on the previous article [9] were used as a basis for searching for more complex SV. By conducting a BLAST [28] search for HNF1 binding sites and an insertion of unknown origin “GAAGAGCTCAAGCTTTCC” (X-1), which was discovered in the previous study, 11,430 HBV sequences containing either an HNF1 binding site or X-1 were obtained. The HBV sequences were analyzed using the CLUSTAL W program [29] and repeated manual inspection was performed to find HBV strains with complex SVs. During the analysis, additional insertional motif sequences of unknown origin, “GGGCCGAACCAGA” (X-2), and “TCTTATGTAAGAGG” (X-3), were identified. A further BLAST search for X-2 and X-3 yielded 14,542 HBV sequences containing insertions of either X-2 or X-3. These HBV sequences also were analyzed using CLUSTAL W. From these analyses, 68 candidate HBV strains were identified. Through comparison with the reference sequence (V00866) using CLUSTAL W, the compositions of the complex SVs were further analyzed using BLAST searches and visual inspection, and finally, 55 HBV strains were confirmed to harbor complex SVs, shown as Nos. 16 to 70 in Additional file 1: Table S1 and Additional file 2: Table S2, Fig. 1a-f, and Additional file 3: Figure S1A-AM. During the initial search, “TCTTATGTAAGAGG” (X-3) was considered to be an insertion of unknown origin; however, during the second analysis, it was found that this sequence motif is complementary to part of box α in the core upstream of regulatory sequences (CURS)/enhancer II. Information on methods used in the determination of HBV genetic sequences were searched, and are shown in Additional file 2: Table S2.

### Positions of complex SVs observed in HBV genetic sequences

**Fig. 2:**
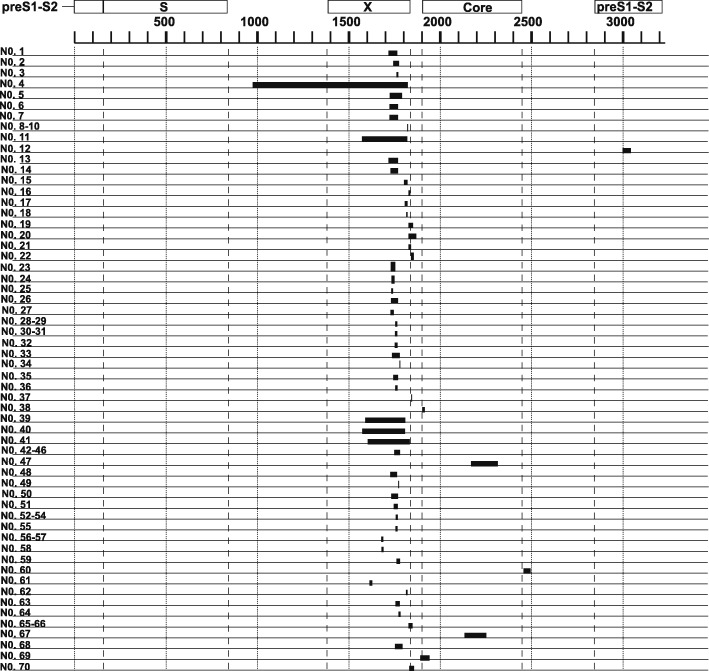
Positions of complex SVs in the HBV nucleotide sequence. Positions where complex SVs were observed in HBV genomes were analyzed. Each black rectangle shows the position and length of complex SVs

The locations of the complex SVs in the genetic sequence of HBV are shown in Fig. 2. In 66/70 (94.3%) HBV strains, complex SVs were observed in the region nt 1500–2000, containing the X ORF and pre-Core/Core ORF. Nt 1500–2000 also contains the CURS/basic core promoter (BCP) and enhancer II, regions regulating the transcription of the HBV genome.

### Changes in genetic sequence identity caused by complex SVs

**Fig. 3:**
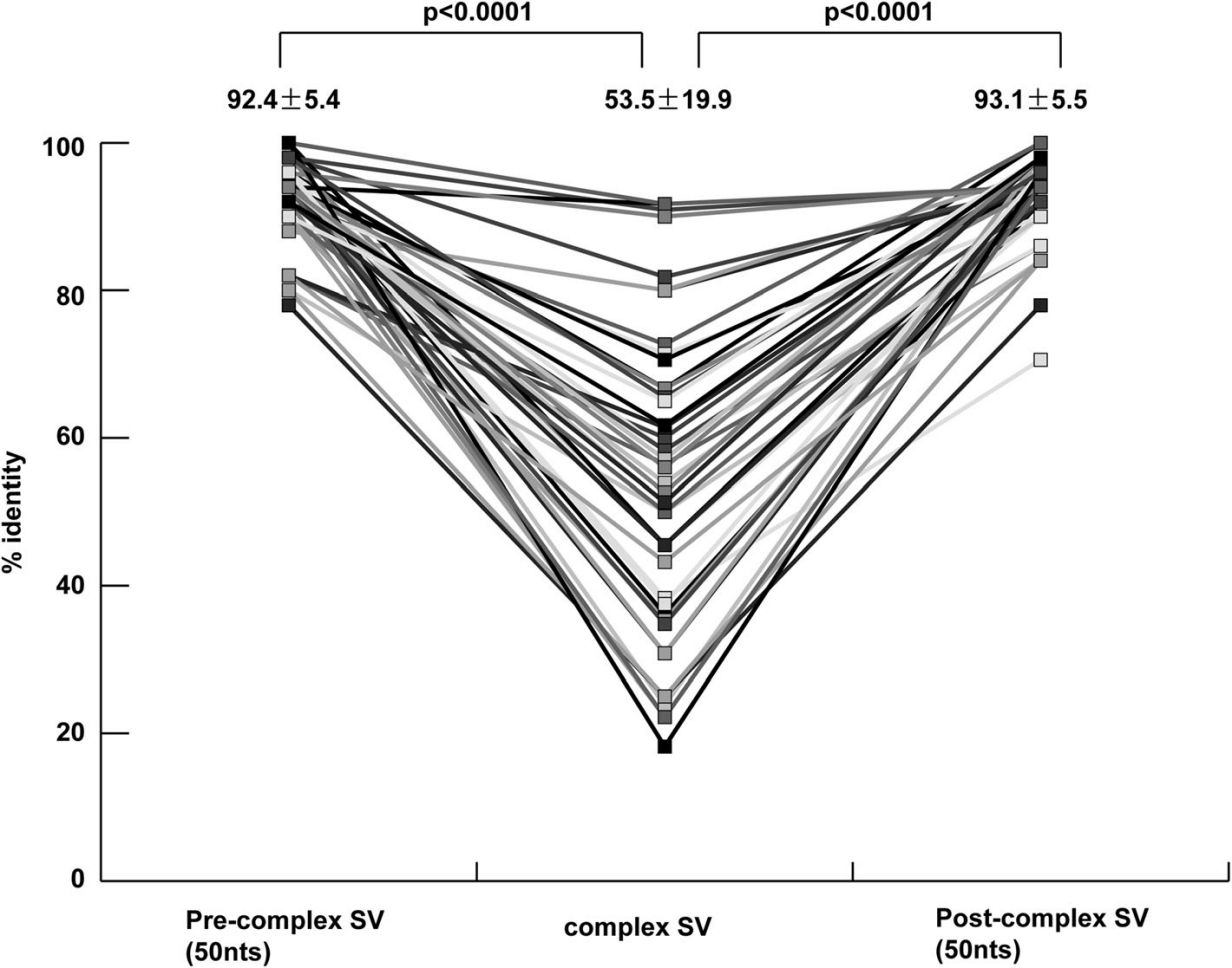
Comparison of sequence identities in the pre-complex SV region (50 nts), complex SV region, and post-complex SV region (50 nts). Percent identities between strains with complex SVs and the reference sequence (V00866) were analyzed by MAFFT [30] for pre-complex SV regions, complex SV regions, and post-complex SV regions

Unlike point mutations, complex SVs cause drastic genetic changes to HBV genome sequences, as shown in Fig. 1a-f, and Additional file 3: Figure S1A-AM. Therefore, changes in genetic sequence identity caused by complex SVs were compared with those caused by nucleotide substitutions. First, the genetic sequence identities of 50 nts located at the 5′ side of complex SV sites were examined using MAFFT [30] with reference sequence (V00866); second, those of the complex SV sites were examined. Then, the 50 nts located at the 3′ side of complex SV sites were examined. Fifty-one HBV strains with sufficient genetic sequence available for comparison were analyzed. As shown in Fig. 3, the genetic sequence identity in the complex SV sites was significantly lower than the pre- and post-complex SV sites. Genetic changes in the pre- and post-complex SV sites reflected nucleotide substitutions.

### Characteristics of complex SVs in HBV

The characteristics of complex SVs in HBV were investigated. The genotypes of the HBV strains with complex SVs were analyzed. Phylogenetic analysis was performed to determine the genotypes of 44 strains for which complete genome sequences were available (Additional file 4: Figure S2). The genotypes of the HBV strains with partial sequences were also determined by phylogenetic analyses. The proportions of HBV genotypes C (30.0%), D (20.0%), and B (20.0%) were much higher than other genotypes. Five were recombinant strains, and three were C/D recombinants, and two were D/E recombinants as shown in Additional file 1: Table S1. Next, sequence gaps in the rearrangements were analyzed. Sequence gaps were found in 56/70 (80.0%) of strains with complex SVs, as shown in Table 1. The average numbers of nucleotide acquired by insertion and/or duplication, and deleted were 36.7 nts, and 45.7 nts, respectively. On the other hand, approximately 20% of HBV strains with complex SVs showed no gaps and the variations were mainly caused by insertions and deletions of the same length. Regarding the composition of complex SVs, the frequency of insertions, and deletions was much higher than duplications (92.9 and 91.4% vs. 25.7%). In addition, the frequency of insertion types was analyzed. Approximately 50% of insertions featured the HNF1 binding site, followed by an insertion of unknown origin (X-1) and a sequence complementary to part of box α in enhancer II. Interestingly, one sequence motif, which initially was considered as an insertion of unknown origin (X-3), turned out to be complementary to the partial box α in enhancer II. Genetic sequences of insertional motifs in HBV strains analyzed in this study, such as HNF1 binding site, insertion of unknown origin (X-1), insertion of unknown origin (X-2), and partial box α in enhancer II, are shown in Additional file 5: Figure S3A to S3D, respectively. Eleven miscellaneous insertions contained part of BCP region (nt 1742 to 1849) (*n* = 7), pre-S1 promoter (3′ side of HNF1 binding site) to pre-S1 ORF (nt 2800–3000) (*n* = 2), area close to enhancer I region (nt 967–1002) (*n* = 1), and insertion of unknown origin “GATGCGACA” (*n* = 1). Clinically, at least 11 complex SVs were observed in patients with HCC (Additional file 1: Table S1).

**Table 1:** Characteristics of complex SVs in HBV

Sequence gap in complex SVs	56/70 (80.0%)
SVs in complex SVs	
Insertion (+)	65/70 (92.9%)
Deletion (+)	64/70 (91.4%)
Duplication (+)	18/70 (25.7%)
Types of insertion	(*n* = 65)
A. HNF1 binding site	32 (49.2%)
B. Insertion of unknown origin (X-1)	12 (18.5%)
C. Insertion of unknown origin (X-2)	4 (6.2%)
D. Sequence complementary to part of box α in enhancer II	6 (9.2%)
E. Miscellaneous	11 (16.9%)

*SV* structural variation, *HNF1* hepatocyte nuclear factor 1

Then, possible association between complex SVs and BCP/precore (PC) mutations was analyzed. Regarding BCP mutation, BCP mutation points (A1762T/G1764A) were included in complex SV sites in 40/70 of HBV strains with complex SVs. In 30 strains with BCP mutation status available, BCP mutation pattern was not associated with specific complex SV pattern. Regarding PC mutation, 49/70 were PC wild. 17/70 were PC mutant. In two, information of PC was not available. In two, complex SVs were observed in PC site. PC mutation pattern was not associated with specific complex SV pattern. For insertional motif sequence, PC wild type had higher frequency of HNF1 binding site insertion than PC mutant type (*p* = 0.006). In addition, PC mutation type had higher frequency of insertion of unknown origin (X-1) than PC wild type (*p* = 0.006).

### Classification of complex SVs in HBV

Seventy HBV strains with complex SVs were classified provisionally according to their SV patterns in order to elucidate the number of patterns of complex SVs and clarify the combination of highest frequency. As shown in Table 2, they were classified into six groups, insertion (+)/deletion (+) (class I), insertion (+)/deletion (+)/duplication (+) (class II), insertion (+)/duplication (+) (class III), deletion (+)/duplication (+) (class IV), multiple duplications (Class V), and highly complicated (class VI), which contained four or more SVs. Further, insertions were divided into five patterns. Insertions (+)/deletion (+) (class I) (70.0%) were more common than other patterns. In addition, in class I (insertion (+)/deletion (+)), HNF1 binding site insertion was much more common than other insertions. Class I insertions with the HNF1 binding site were observed in 24/70 (34.3%) of the HBV strains with complex SVs.

**Table 2:** Classification of HBV strains with complex SVs

	(*n* = 70)
I. Insertion (+), Deletion (+)	49 (70.0%)
Types of Insertion	
A. HNF1 binding site	24 (34.3%)
B.Insertion of unknown origin (X-1)	7 (10.0%)
C. Insertion of unknown origin (X-2)	3 (4.3%)
D. Sequence complementary to part of box α in enhancer II	6 (8.6%)
E. Miscellaneous	9 (12.9%)
II. Insertion (+), Deletion (+), Duplication (+)	6 (8.6%)
Types of Insertion	
A. HNF1 binding site	4 (5.7%)
B. Insertion of unknown origin (X-1)	1 (1.4%)
C. Insertion of unknown origin (X-2)	0 (0.0%)
D. Sequence complementary to part of box α in enhancer II	0 (0.0%)
E. Miscellaneous	1 (1.4%)
III. Insertion (+), Duplication (+)	5 (7.1%)
Types of Insertion	
A. HNF1 binding site	2 (2.9%)
B. Insertion of unknown origin (X-1)	3 (4.3%)
C. Insertion of unknown origin (X-2)	0 (0.0%)
D. Sequence complementary to part of box α in enhancer II	0 (0.0%)
E. Miscellaneous	0 (0.0%)
IV. Deletion (+), Duplication (+)	4 (5.7%)
V. Duplications (+)	1 (1.4%)
VI.Highly complicated (four or more SVs)	5 (7.1%)

Insertion patterns are shown in order of discovery. Some HBV strains with two SVs of identical pattern and one other SV are included in group I, III, IV. SV, structural variation

*HNF1* hepatocyte nuclear factor 1

### Comparison of complex SV strains with and without HNF1binding site insertion

Our data showed that HNF1binding site insertion was the main insertional motif sequence in complex SVs, and more information of complex SVs with HNF1 binding site may provide clues to the mechanisms of complex SV. Therefore, background differences between complex SVs with HNF1 binding site insertion and those without HNF1 binding site insertion were searched (Table 3). For HBV genotypes, complex SVs with HNF1 binding site insertion were limited to HBV/B to HBV/E. On the other hand, those without HNF1 binding site insertion contained HBV/A, HBV/F, HBV/H, and HBV/I, in addition to HBV/B to HBV/D. Strains with HBV/E were not observed in those without HNF1 binding site. For regional origins, complex SVs with HNF1 binding site insertion were observed more frequently in the Middle East than those without HNF1 binding site insertion, and only those without HNF1 binding site insertion were observed in Central and South America. For the locations in HBV genome, complex SVs with HNF1 binding site insertion were limited in nt 1500–2000. For classes of complex SVs, no clear differences were observed.

**Table 3:** Comparison between Complex SVs with and without HNF1 insertion

	Complex SV with HNF1 insertion (*n* = 32)	Complex SV without HNF1 insertion (*n* = 38)
Genotype		
A	0	4
B	7	7
C	12	9
D	8	6
E	4	0
F	0	4
H	0	2
I	0	1
miscellaneous	1	5
Region		
Asia	18	21
Europe	3	6
Africa	3	4
Middle East	8	1
C. and S. America	0	6
Location		
nt 1500–2000	32	34
nt 1–1499, 2001-	0	4
SV patterns (Class in Table 2)		
I	24	25
II	4	2
III	2	3
IV	0	4
V	0	1
VI	2	3

*SV* structural variation, *HNF1* hepatocyte nuclear factor 1, *C* Central, *S* South

Then, we searched specific association between complex SVs and particular genotypes. No association between complex SV classes and HBV genotypes was found. Regarding insertional motif sequences, all the recombinant HBV strains contained either insertion of unknown origin (X-1) or complementary sequence of insertion of unknown origin (X-1). In addition, 5 of 6 HBV strains with insertion of complementary sequence of part of box α in enhancer II were genotypes F or H, which are distributed in South America. Furthermore, insertion of unknown origin (X-2) was limited to genotypes B and C.

## Discussion

The concept of this study began with a case report of unusual HBV genetic rearrangement, which featured a combination of deletion, insertion and duplication [27]. At that time, genetic rearrangements of this kind had not been defined. More recent studies have defined complex SVs that cannot be classified by canonical genetic changes [22–25]. Yalcin et al. [24] described complex SVs as two or more structural variants co-occurring at the same locus. By adapting the concept of complex SVs in human and mouse genomes, complex SVs in HBV were analyzed for the first time in the previous report [9]. In this report, 55 HBV strains with complex SVs were identified, in addition to 15 HBV strains in the previous report, and a total of 70 HBV strains were used in the analysis.

Regarding their positions in HBV genome, 94.3% of complex SVs were located between nt 1500 and 2000, the region containing the X and pre-C/Core ORFs, as shown in Fig. 2. Regulatory sequence of transcription such as CURS/BCP and enhancer II, also are located in this region. The HNF1 binding site, which originated from the pre-S1 promoter [31], was the most common insertional motif in complex SVs (Table 1 and Additional file 5: Figure S3A). The HNF1 binding site is very important for the transcription of pre-S1 mRNA [32]. Canonical simple insertion of the HNF1 binding site can affect the transcription of pregenomic RNA [33]. Simple HNF1 binding site insertion in the BCP has been reported in fulminant hepatitis and hepatic failure [33, 34]. In this study, a novel insertional motif was discovered and found to be complementary to box α in CURS/enhancer II, as shown in Table 1 and Additional file 5: Figure S3D. Box α in enhancer II also affects transcription of HBV genome. Yuh et al. [35] reported that box α is one of several elements controlling BCP activity and can enhance that activity by more than 100-fold. Duplication of box α has been reported in previous studies [36]. Furthermore, data from previous studies and herein show that both the HNF1 binding site and box α may be inserted as either canonical SVs, such as insertion or duplication, or as complex SVs. The reason why these motif sequences modify HBV genetic sequences in certain HBV strains is not clear, and further investigation is required to elucidate their role.

The virological significance of complex SVs in HBV life cycle was analyzed in previous studies. In in vitro study using complex SV strain No. 1, construct with complex SVs replicated much more than a wild type construct. Northern blot analysis showed the construct with complex SVs had higher pregenomic and preS/S RNA levels [27]. In addition, localization of HBcAg in nucleus and perinucleus was observed, which was compatible with histopathological data of the patient. Another in vitro study using complex SV strain No. 6 showed similar data. The complex SV strain had higher pregenomic and preS/S RNA levels [36]. Regarding HBcAg expression in cells, Pult et al. [33] reported that accumulation of massive amount of cytoplasmic and nuclear HBcAg in infected cells has cytopathic effect. Excessive production and specific localization of viral protein may be associated with pathology of HBV harboring complex SVs. Clinically, at least 11, 2, and 2 strains were observed in patients with HCC, fulminant hepatitis patients, and patients with severe chronic liver disease, respectively. It is highly possible that strains with complex SVs affect transcription of HBV genome by modulating transcriptional factor binding sites and production of HBV-related proteins, and are related to severe liver disease such as fulminant hepatitis or HCC.

In this study, the impact of complex SVs on HBV identity was analyzed (Fig. 3). Until now, genetic sequence identity between HBV strains has been considered to be caused by nucleotide substitutions and recombinations. Differences in genotypes, defined by Okamoto et al. [3] as 8% nucleotide differences, reflect accumulation of nucleotide substitutions. In this study, the changes in genetic identity shown in Fig. 3 reflect a change completely different from nucleotide substitutions, as shown in Fig. 1a-f, Additional file 3: Figure S1A-AM. Genetic identities in pre-complex SVs and post-complex SVs reflect the accumulation of nucleotide substitutions between the reference sequence (Genotype A, V00866) and each strains with complex SVs. These reflect inter- and intra-genotypic nucleotide identity, with approximately 70.0 to 100% identity.

It is fairly difficult to detect complex SVs of HBV. However, the characteristics of HBV with complex SVs support detection of those strains with such rearrangements. First, 56/70 (80.0%) of strains with complex SVs showed sequence gaps, as illustrated in Table 1 and detecting sequence gaps is a methods for finding complex SVs of HBV. On the other hand, approximately 20% of HBV strains with complex SVs had no gaps and were mostly caused by insertions and deletions of same nucleotide length; in these cases, a BLAST search for insertional motifs is important. Furthermore, analysis of sequence identity showed that low sequence identity was observed in the area with complex SVs, as shown in Fig. 3. Therefore, sequence gaps, insertional sequence motifs, and low sequence identity are important for finding these variant strains.

A provisional classification of complex SVs of HBV was carried out, based on the types of SVs forming complex SVs in order to clarify the number of variations and the pattern with highest frequency. In this analysis, HBV strains were categorized into six groups, then subgroups were defined in cases with insertions. The predominant pattern of insertion and deletion (class I) with HNF1 insertion was clarified. Further, comparison between complex SVs with and without HNF1 insertion revealed that those with HNF1 insertions were observed only in genotypes B to E. The result may suggest that certain genetic structure is required for the rearrangement to occur.

## Conclusion

We have identified new HBV strains with complex SVs. A total of 70 HBV strains with complex SVs were analyzed, and characteristics of complex SVs in HBV were clarified in this study.

## Methods

### Article search

Articles in PubMed were searched for additional HBV strains with complex SVs using the keywords (“HBV” and “mutation”); (“HBV” and “recombination”); (“HBV” and “insertion”); (“HBV” and “deletion”); (“HBV” and “rearrangement”) or (“HBV” and “duplication”). Articles published between January and December 2017 were searched, earlier articles having been searched previously [9].

### Analysis of complex SVs

Complex SVs are defined as SVs with multiple breakpoints and comprise a complex mixture of deletions, insertions, and duplications [23, 24]. The candidate genetic sequences harboring complex SVs were analyzed using the CLUSTAL W program [29], and alignments with the reference sequence (HBV/A, V00866) were created. When partial sequences with low similarity to the reference sequences and/or sequence gaps were observed by visual inspection, a similarity search for the unique sequence was conducted using NCBI BLAST 2.2.31. [28], and then additional analysis with visual inspection was performed by emulating the patterns of complex SVs in previous articles as references [23, 24]. Figures along with nucleotide alignments show each complex SV pattern and these were analyzed in details (Fig. 1a-f). Furthermore, four insertional motif sequences, an HNF1 binding site, and insertions of unknown origins, X-1 to X-3, were directly searched in NCBI BLAST [28] and the HBV genetic sequences retrieved were analyzed for complex SVs. HBV genomes integrated into human genomes were not included in this study.

### Reference sequences

Using the CLUSTAL W software program [29], consensus reference sequences of HBV/A to E were analyzed. 150, 40, 168, 79, and 38 complete genome sequences of HBV/A, HBV/B, HBV/C, HBV/D, and HBV/E, respectively, were used to determine these consensus sequences, as described previously [9].

### Phylogenetic analysis and recombination analysis

The MEGA software version 6 [37] was used to perform phylogenetic analyses, using the neighbor-joining method. Bootstrap resampling and reconstruction with 1000 replicates were performed. Genetic distance calculation and pairwise distance comparisons were performed using the Kimura Two-parameter model integrated into the MEGA software. Inter-genotype recombination of HBV strains was searched for using the SIMPLOT program version 3.5.1 [38].

### Percent identity analysis

Percent identities between HBV strains with complex SVs and reference HBV genome sequence of V00866 were analyzed by MAFFT [30].

### Statistical analysis

The paired t-test was used to compare the percent identity of genetic sequences between the pre-complex SV region and complex SV region, and between the complex SV region and post-complex SV region. Statistical analyses were performed using STATA 8.1 (College Station, TX). A *P*-Value of < 0.05 was considered statistically significant.

## Additional files

Additional file 1: **Table S1.** HBV strains with complex SVs. (DOCX 25 kb)

Additional file 2: **Table S2.** Method of genetic sequencing. (DOCX 29 kb)

Additional file 3: **Figure S1.** Patterns of complex SVs in HBV strains. (DOCX 929 kb)

Additional file 4: **Figure S2.** HBV genotypes. (DOCX 54 kb)

Additional file 5: **Figure S3.** Insertional motif genetic sequences. (DOCX 93 kb)

## Abbreviations

BCP: Basic core promoter
CURS: Core upstream of regulatory sequences
HBsAg: Hepatitis B surface antigen
HBV: Hepatitis B virus
HNF1: Hepatocyte nuclear factor 1
NA: Nucleos(t)ide analogue
nt: Nucleotide(s)
SV: Structural variation
