# Supplementary material for: Characterization of hepatitis B virus with complex structural variations
Source: BMC Microbiol. 2018 Dec 3;18:202. doi: 10.1186/s12866-018-1350-1 (PMC6276219; doi:10.1186/s12866-018-1350-1)
Supplement: Supplementary file 1 — Table S1. HBV strains with complex SVs. (DOCX 25 kb) [file 12866_2018_1350_MOESM1_ESM.docx]

**Table S1. HBV strains with complex SVs**

| Strain No. | Accession No. | Country/ region  of origin | Sequence  gap (+/-) | Gt | BCP/PC status | Clinical disease | Pattern of complex SV |
| --- | --- | --- | --- | --- | --- | --- | --- |
| 1 | AB219530 | Nigeria | + | E | Complex SV/Wild | Severe CH | Ins+Del+Dup |
| 2 | AM494791 | Central Africa | + | E | Complex SV/Wild | Unknown | Ins+Del+Dup |
| 3 | NA (CSV3/Pt8.29) | France | + | ND | Complex SV/NA | Unknown | Ins+Del |
| 4 | DQ890380 | Korea | + | C | Complex SV/Wild | HCC | Ins+Del |
| 5 | NA (CSV5/I) | Germany | + | A | Complex SV/Wild | Severe CLD | Del+Dup |
| 6 | NA (CSV6/G) | Germany | + | A | Mutant/NA | LC | Del+Dup |
| 7 | FN594754 | Niger | + | E | Complex SV/Wild | ASC | Ins+Del |
| 8 | FN594768 | Niger | + | D/E | Mutant/Mutant | ASC | Ins+Dup |
| 9 | AB675682 | China | + | C/D | Wild/Wild | Unknown | Ins+Dup |
| 10 | AB675681 | China | + | C/D | Wild/Wild | Unknown | Ins+Dup |
| 11 | DQ336691 | Italy | + | D | Complex SV/Mutant | HCC | Ins+Del+Dup |
| 12 | FR714499 | China | + | I | Wild/Wild | Unknown | Ins+Del |
| 13 | KR811830 | France | + | D | Complex SV/Wild | HCC | Ins+Del+Dup |
| 14 | AB915179 | Saudi Arabia | + | E | Complex SV/Wild | Unknown | Ins+Del+Dup |
| 15 | JN664924 | India | - | C/D | Wild/Mutant | Chr carrier | Ins+Del |
| 16 | KU711666 | Ghana | - | D/E | Wild/Mutant | HIV(+),  Chr carrier | Ins+Del |
| 17 | KP718105 | Panama | - | F | Wild/Wild | Unknown | Ins+Del |
| 18 | EU717213 | China | + | C | Mutant/Mutant | Unknown | Ins+Del |
| 19 | KU964137 | China | - | B | Wild/Mutant | Unknown | Ins+Del |
| 20 | KM359477 | Taiwan | + | C | Wild/Wild | Unknown | Ins+Del |
| 21 | KM392078 | Taiwan | + | B | Wild/Complex SV | Unknown | Ins+Del |
| 22 | KF922422 | South Africa | + | A | Wild/Wild | Unknown | Ins+Del |
| 23 | AB976562 | Indonesia | ~~-~~ | B | Complex SV/Mutant | Unknown | Ins+Del |
| 24 | KJ949577 | China | + | B | Complex SV/Wild | CH | Ins+Del |
| 25 | KJ949267 | China | + | C | Complex SV/Wild | CH | Ins+Del |
| 26 | KC774350 | China | - | C | Complex SV/Wild | ASC | Highly complicated |
| 27 | KC774357 | China | - | C | Mutant/Wild | ASC | Ins+Del |
| 28 | KJ173418 | China | - | B | Complex SV/Wild | Unknown | Ins+Del |
| 29 | KJ173419 | China | - | B | Complex SV/Wild | Unknown | Ins+Del |
| 30 | KF165850 | China | + | C | Complex SV/Wild | Unknown | Ins+Del |
| 31 | KM875415 | China | + | C | Complex SV/Wild | HCC | Ins+Del |
| 32 | KF165588 | China | + | B | Complex SV/Wild | Unknown | Ins+Del |
| 33 | JN257184 | Syria | + | D | Complex SV/Wild | Unknown | Highly complicated |
| 34 | GU079050 | China | + | B | Complex SV/Mutant | Unknown | Ins+Dup |
| 35 | EU726948 | Iran | + | D | Complex SV/Wild | Unknown | Ins+Del |
| 36 | EU547561 | Malaysia | + | C | Complex SV/Mutant | Unknown | Ins+Del |
| 37 | EU306725 | China | + | C | Mutant/Wild | ASC | 2Dups |
| 38 | AB330366 | Tajikistan | + | D | Mutant/Mutant | CLD | Ins+Del |
| 39 | DQ464179 | Italy | + | D | Complex SV/Mutant | HCC | Ins+Del |
| 40 | DQ464180 | Italy | + | D | Complex SV/Mutant | HCC | Ins+Del |
| 41 | DQ464177 | Italy | + | D | Complex SV/Mutant | HCC | Highly complicated |
| 42 | AY489307 | Yemen | + | D | Complex SV/Wild | Unknown | Ins+Del |
| 43 | AY489308 | Yemen | + | D | Complex SV/Wild | Unknown | Ins+Del |
| 44 | AY489309 | Yemen | + | D | Complex SV/Wild | Unknown | Ins+Del |
| 45 | AY489310 | Yemen | + | D | Complex SV/Wild | Unknown | Ins+Del |
| 46 | AY489312 | Yemen | + | D | Complex SV/Wild | Unknown | Ins+Del |
| 47 | AY206393 | China | + | C | Deletion/Wild | HCC | Del+Dup |
| 48 | AF461362 | China | + | B | Complex SV/Wild | Fulminant hepatitis | Ins+Del+Dup |
| 49 | X98075 | Germany | + | B | Wild/Wild | Fulminant  Hepatitis | Ins+Dup |
| 50 | KJ173027 | China | + | C | Complex SV/Wild | Unknown | Ins+Del |
| 51 | KF165702 | China | + | B | Complex SV/Wild | Unknown | Ins+Del |
| 52 | KF165956 | China | - | C | Complex SV/Wild | Unknown | Ins+Del |
| 53 | KF165066 | China | - | C | Complex SV/Wild | Unknown | Ins+Del |
| 54 | KF165010 | China | - | C | Complex SV/Wild | Unknown | Ins+Del |
| 55 | GQ858570 | China | + | C | Complex SV/Wild | Unknown | Ins+Del |
| 56 | AB516394 | Mexico | + | H | Mutant/Wild | Unknown | Ins+Del |
| 57 | KP995099 | Venezuela | + | F | Mutant/Wild | HCC | Ins+Del |
| 58 | AP007261 | Japan | + | H | Mutant/Wild | HIV (+) | Ins+Del |
| 59 | AB674435 | Turkey | + | D | Complex SV/Mutant | Unknown | Ins+Del |
| 60 | AB670257 | Japan | + | C | Wild/Wild | HCC | Ins+Del |
| 61 | JX504531 | China | + | B | Mutant/Mutant | Unknown | Ins+Del |
| 62 | JX507211 | Panama | - | C | Mutant/Wild | Unknown | Ins+Del |
| 63 | KF167055 | China | + | C | Complex SV/Mutant | Unknown | Ins+Del |
| 64 | HM214756 | Argentina | + | F | Wild/Wild | Chr carrier | Ins+Del |
| 65 | HM214757 | Argentina | + | F | Wild/Wild | Chr carrier | Ins+Del |
| 66 | AY217374 | China | - | C | Mutant/Wild | Chr carrier | Ins+Del |
| 67 | EU660226 | Taiwan | + | C | Mutant/Mutant | Unknown | Del+Dup |
| 68 | HQ646556 | South Africa | + | A | Complex SV/Wild | HCC | Highly complicated |
| 69 | KM392078 | Taiwan | + | B | Wild/Complex SV | Unknown | Highly complicated |
| 70 | KM392071 | Taiwan | + | B | Wild/Wild | Unknown | Ins+Del |

Information on Nos. 1 to 15 is obtained from the previous article. ^1^ Nos. 21 and 69, are from the same strain (KM392078) and, in this article, two complex SVs were considered as separate cases. In addition, No. 39 and 40, which were different complex SVs from the same strain, were also considered as separate cases, and total number was 70. CSV3/Pt8.29, CSV5/I, and CSV6/G correspond to HBV strains CSV3/Pt8.29, CSV5/I, and CSV6/G in the previous report.^2^ “/” in Gt indicates recombination. No., number; Gt, genotype; NA, not applicable; CH, chronic hepatitis; ASC, asymptomatic carrier; CLD, chronic liver disease; LC, liver cirrhosis; HCC, hepatocellular carcinoma; Chr, chronic; HIV, human immunodeficiency virus; Ins, insertion; Del, deletion; Dup, duplication.
