# Supplementary material for: Characterization of hepatitis B virus with complex structural variations
Source: BMC Microbiol. 2018 Dec 3;18:202. doi: 10.1186/s12866-018-1350-1 (PMC6276219; doi:10.1186/s12866-018-1350-1)
Supplement: Supplementary file 2 — Table S2. Method of genetic sequencing. (DOCX 29 kb) [file 12866_2018_1350_MOESM2_ESM.docx]

**Table S2 Method of genetic sequencing**

| Strain No. | Method of genetic sequencing | Major/Minor clone | Number of clones  with complex SVs (%) | Ref |
| --- | --- | --- | --- | --- |
| 1 | Cloning-sequencing | Major clone | 13/19 (68.4) | Fujiwara et al. (2005)^3^ |
| 2 | Cloning-sequencing | data not available | data not available | Bekondi et al. (2007)^4^ |
| 3 | Cloning-sequencing | Minor clone | 1/7 (14.3) | Parekh et al. (2003)^5^ |
| 4 | Cloning-sequencing | Major clone | 3/3 (100) | Kim et al. (2007)^6^ |
| 5 | Cloning-sequencing | Minor clone | 5/12 (41.7) | Gunther et al. (1996)^2^ |
| 6 | Cloning-sequencing | Minor clone | 1/17 (5.9) | Gunther et al. (1996)^2^ |
| 7 | Direct sequencing |  |  | Abdou et al. (2010)^7^ |
| 8 | Direct sequencing |  |  | Abdou et al. (2010)^7^ |
| 9 | Cloning-sequencing | Major clone | 18/24 (75.0) |  |
| 10 | Cloning-sequencing | Major clone | 10/20 (50.0) |  |
| 11 | Cloning-sequencing | Minor clone | 1/8 (12.5) | Pollicino et al.(2007)^8^ |
| 12 | Direct sequencing |  |  | Fang et al. (2011)^9^ |
| 13 | Cloning-sequencing | Major Clone | 23/23 (100) |  |
| 14 | Direct sequencing |  |  |  |
| 15 | Direct sequencing |  |  | Ghosh et al. (2013)^10^ |
| 16 | NGS | consensus |  | Boyce et al. (2017)^11^ |
| 17 | Direct sequencing |  |  | Martinez et al.(2015)^12^ |
| 18 | Data not available |  |  |  |
| 19 | Cloning-sequencing | Data not available | Data not available | Xue et al. (2016)^13^ |
| 20 | Data not available |  |  |  |
| 21 | Data not available |  |  |  |
| 22 | Direct sequencing |  |  | Mayaphi et al.(2013)^14^ |
| 23 | Direct sequencing |  |  | Utsumi et al. (2015)^15^ |
| 24 | Cloning-sequencing | data not available | data not available | Peng et al. (2015)^16^ |
| 25 | Cloning-sequencing | data not available | data not available | Peng et al. (2015)^16^ |
| 26 | Cloning-sequencing | data not available | data not available | Chen et al. (2014)^17^ |
| 27 | Cloning-sequencing | data not available | data not available | Chen et al. (2014)^17^ |
| 28 | Cloning-sequencing | data not available | data not available | Li et al. (2014)^18^ |
| 29 | Cloning-sequencing | data not available | data not available | Li et al. (2014)^18^ |
| 30 | Data not available |  |  |  |
| 31 | Direct sequencing |  |  | Zhang et al. (2016)^19^ |
| 32 | Data not available |  |  |  |
| 33 | Direct sequencing |  |  | Habbal et al. (2012)^20^ |
| 34 | Data not available |  |  |  |
| 35 | Direct sequencing |  |  | Mohebbi et al.(2008)^21^ |
| 36 | Data not available |  |  |  |
| 37 | Cloning-sequencing | Minor clone | 1/9 (11.1) | Shen et al. (2008)^22^ |
| 38 | Direct sequencing |  |  | Khan et al. (2008)^23^ |
| 39 | Cloning-sequencing | Minor clone | 1/5 (20.0) | Pollicino et al. (2007)^8^ |
| 40 | Cloning-sequencing | Minor clone | 2/5 (40.0) | Pollicino et al. (2007)^8^ |
| 41 | Cloning-sequencing | Minor clone | 2/5 (40.0) | Pollicino et al. (2007)^8^ |
| 42 | Direct sequencing |  |  | Sallam et al. (2004)^24^ |
| 43 | Direct sequencing |  |  | Sallam et al. (2004)^24^ |
| 44 | Direct sequencing |  |  | Sallam et al. (2004)^24^ |
| 45 | Direct sequencing |  |  | Sallam et al. (2004)^24^ |
| 46 | Direct sequencing |  |  | Sallam et al. (2004)^24^ |
| 47 | Data not available |  |  |  |
| 48 | Data not available |  |  |  |
| 49 | Cloning-sequencing | Minor clone | 2% | Pult et al. (1997)^25^ |
| 50 | Cloning-sequencing | data not available | data not available | Li et al. (2014)^18^ |
| 51 | Data not available |  |  |  |
| 52 | Data not available |  |  |  |
| 53 | Data not available |  |  |  |
| 54 | Data not available |  |  |  |
| 55 | Direct sequencing |  |  | Liu et al. (2011)^26^ |
| 56 | Direct sequencing |  |  | Roman et al. (2010)^27^ |
| 57 | Direct sequencing |  |  | Puche et al. (2016)^28^ |
| 58 | Cloning-sequencing | Major clone | Data not available | Shibayama et al. (2005)^29^ |
| 59 | Direct sequencing |  |  | Sunbul et al. (2013)^30^ |
| 60 | Direct sequencing |  |  | Tatsukawa et al. (2011)^31^ |
| 61 | Data not available |  |  |  |
| 62 | Direct sequencing |  |  | Martinez et al.(2013)^32^ |
| 63 | Data not available |  |  |  |
| 64 | Direct sequencing |  |  | Pezzano et al. (2011)^33^ |
| 65 | Direct sequencing |  |  | Pezzano et al. (2011)^33^ |
| 66 | Cloning-sequencing | Data not available | Data not available | Luo et al. (2004)^34^ |
| 67 | Data not available |  |  |  |
| 68 | Cloning-sequencing | Major clone | 5/5 (100) | Skelton et al. (2012)^35^ |
| 69 | Data not available |  |  |  |
| 70 | Data not available |  |  |  |

Cloning-sequencing, the genetic sequencing was performed after molecular cloning. NGS, next generation sequencing

**References to Supplementary materials**

1 Fujiwara, K., Matsunami, K., Iio, E., Nojiri, S., Joh, T., 2017. Novel non-canonical genetic rearrangements termed "complex structural variations" in HBV genome. Virus Res 238, 84-93.

2 Gunther, S., Piwon, N., Iwanska, A., Schilling, R., Meisel, H., Will, H., 1996. Type, prevalence, and significance of core promoter/enhancer II mutations in hepatitis B viruses from immunosuppressed patients with severe liver disease. J Virol 70(12), 8318-8331.

3 Fujiwara, K., Tanaka, Y., Paulon, E., Orito, E., Sugiyama, M., Ito, K., Ueda, R., Mizokami, M., Naoumov, N.V., 2005. Novel type of hepatitis B virus mutation: replacement mutation involving a hepatocyte nuclear factor 1 binding site tandem repeat in chronic hepatitis B virus genotype E. J Virol 79(22), 14404-14410.

4 Bekondi, C., Olinger, C.M., Boua, N., Talarmin, A., Muller, C.P., Le Faou, A., Venard, V., 2007. Central African Republic is part of the West-African hepatitis B virus genotype E crescent. J Clin Virol 40(1), 31-37.

5 Parekh, S., Zoulim, F., Ahn, S.H., Tsai, A., Li, J., Kawai, S., Khan, N., Trepo, C., Wands, J., Tong, S., 2003. Genome replication, virion secretion, and e antigen expression of naturally occurring hepatitis B virus core promoter mutants. J Virol 77(12), 6601-6612.

6 Kim, H., Jee, Y., Mun, H.S., Park, J.H., Yoon, J.H., Kim, Y.J., Lee, H.S., Hyun, J.W., Hwang, E.S., Cha, C.Y., Kook, Y.H., Kim, B.J., 2007. Characterization of two hepatitis B virus populations in a single Korean hepatocellular carcinoma patient with an HBeAg-negative serostatus: a novel X-Gene-deleted strain with inverted duplication sequences of upstream enhancer site II. Intervirology 50(4), 273-280.

7 Abdou Chekaraou, M., Brichler, S., Mansour, W., Le Gal, F., Garba, A., Deny, P., Gordien, E., 2010. A novel hepatitis B virus (HBV) subgenotype D (D8) strain, resulting from recombination between genotypes D and E, is circulating in Niger along with HBV/E strains. J Gen Virol 91(Pt 6), 1609-1620.

8 Pollicino, T., Raffa, G., Costantino, L., Lisa, A., Campello, C., Squadrito, G., Levrero, M., Raimondo, G., 2007. Molecular and functional analysis of occult hepatitis B virus isolates from patients with hepatocellular carcinoma. Hepatology 45(2), 277-285.

9 Fang, Z.L., Hue, S., Sabin, C.A., Li, G.J., Yang, J.Y., Chen, Q.Y., Fang, K.X., Huang, J., Wang, X.Y., Harrison, T.J., 2011. A complex hepatitis B virus (X/C) recombinant is common in Long An county, Guangxi and may have originated in southern China. J Gen Virol 92(Pt 2), 402-411.

10 Ghosh, S., Banerjee, P., Deny, P., Mondal, R.K., Nandi, M., Roychoudhury, A., Das, K., Banerjee, S., Santra, A., Zoulim, F., Chowdhury, A., Datta, S., 2013. New HBV subgenotype D9, a novel D/C recombinant, identified in patients with chronic HBeAg-negative infection in Eastern India. J Viral Hepat 20(3), 209-218.

11 Boyce, C.L., Ganova-Raeva, L., Archampong, T.N.A., Lartey, M., Sagoe, K.W., Obo-Akwa, A., Kenu, E., Kwara, A., Blackard, J.T., 2017. Identification and comparative analysis of hepatitis B virus genotype D/E recombinants in Africa. Virus Genes 53(4), 538-547.

12 Martinez, A.A., Zaldivar, Y., Arteaga, G., de Castillo, Z., Ortiz, A., Mendoza, Y., Castillero, O., Castillo, J.A., Cristina, J., Pascale, J.M., 2015. Phylogenetic Analysis of Hepatitis B Virus Genotypes Circulating in Different Risk Groups of Panama, Evidence of the Introduction of Genotype A2 in the Country. PLoS One 10(7), e0134850.

13 Xue, Y., Wang, M.J., Huang, S.Y., Yang, Z.T., Yu, D.M., Han, Y., Zhu, M.Y., Huang, D., Zhang, D.H., Gong, Q.M., Zhang, X.X., 2016. Characteristics of CpG Islands and their quasispecies of full-length hepatitis B virus genomes from patients at different phases of infection. Springerplus 5(1), 1630.

14 Mayaphi, S.H., Martin, D.J., Mphahlele, M.J., Blackard, J.T., Bowyer, S.M., 2013. Variability of the preC/C region of hepatitis B virus genotype A from a South African cohort predominantly infected with HIV. J Med Virol 85(11), 1883-1892.

15 Utsumi, T., Wahyuni, R.M., Lusida, M.I., Yano, Y., Priambada, N.P., Amin, M., Purwono, P.B., Istimagfiroh, A., Soetjipto, Brule, A., Hotta, H., Hayashi, Y., 2015. Full genome characterization and phylogenetic analysis of hepatitis B virus in gibbons and a caretaker in Central Kalimantan, Indonesia. Arch Virol 160(3), 685-692.

16 Peng, Y., Liu, B., Hou, J., Sun, J., Hao, R., Xiang, K., Yan, L., Zhang, J., Zhuang, H., Li, T., 2015. Naturally occurring deletions/insertions in HBV core promoter tend to decrease in hepatitis B e antigen-positive chronic hepatitis B patients during antiviral therapy. Antivir Ther 20(6), 623-632.

17 Chen, X., Gao, J., Ji, Z., Zhang, W., Zhang, L., Xu, R., Zhang, J., Li, F., Li, S., Hu, S., Shang, L., Shao, Z., Yan, Y., 2014. A description of the hepatitis B virus genomic background in a high-prevalence area in China. Virol J 11, 101.

18 Li, Z., Xie, Z., Ni, H., Zhang, Q., Lu, W., Yin, J., Liu, W., Ding, Y., Zhao, Y., Zhu, Y., Pu, R., Zhang, H., Dong, H., Fu, Y., Sun, Q., Xu, G., Cao, G., 2014. Mother-to-child transmission of hepatitis B virus: evolution of hepatocellular carcinoma-related viral mutations in the post-immunization era. J Clin Virol 61(1), 47-54.

19 Zhang, A.Y., Lai, C.L., Poon, R.T., Huang, F.Y., Seto, W.K., Fung, J., Wong, D.K., Yuen, M.F., 2016. Hepatitis B virus full-length genomic mutations and quasispecies in hepatocellular carcinoma. J Gastroenterol Hepatol 31(9), 1638-1645.

20 Habbal, W., Monem, F., 2012. Rethinking therapeutic decisions for hepatitis B infection in Syria: insights into molecular monitoring. J Infect Dev Ctries 6(10), 744-747.

21 Mohebbi, S.R., Amini-Bavil-Olyaee, S., Zali, N., Noorinayer, B., Derakhshan, F., Chiani, M., Rostami Nejad, M., Antikchi, M.H., Sabahi, F., Zali, M.R., 2008. Molecular epidemiology of hepatitis B virus in Iran. Clin Microbiol Infect 14(9), 858-866.

22 Shen, T., Yan, X.M., Zou, Y.L., Gao, J.M., Dong, H., 2008. Virologic characteristics of hepatitis B virus in patients infected via maternal-fetal transmission. World J Gastroenterol 14(37), 5674-5682.

23 Khan, A., Kurbanov, F., Tanaka, Y., Elkady, A., Sugiyama, M., Dustov, A., Mizokami, M., 2008. Epidemiological and clinical evaluation of hepatitis B, hepatitis C, and delta hepatitis viruses in Tajikistan. J Med Virol 80(2), 268-276.

24 Sallam, T.A., William Tong, C.Y., 2004. African links and hepatitis B virus genotypes in the Republic of Yemen. J Med Virol 73(1), 23-28.

25 Pult, I., Chouard, T., Wieland, S., Klemenz, R., Yaniv, M., Blum, H.E., 1997. A hepatitis B virus mutant with a new hepatocyte nuclear factor 1 binding site emerging in transplant-transmitted fulminant hepatitis B. Hepatology 25(6), 1507-1515.

26 Liu, S., Xie, J., Yin, J., Zhang, H., Zhang, Q., Pu, R., Li, C., Ni, W., Wang, H., Cao, G., 2011. A matched case-control study of hepatitis B virus mutations in the preS and core promoter regions associated independently with hepatocellular carcinoma. J Med Virol 83(1), 45-53.

27 Roman, S., Tanaka, Y., Khan, A., Kurbanov, F., Kato, H., Mizokami, M., Panduro, A., 2010. Occult hepatitis B in the genotype H-infected Nahuas and Huichol native Mexican population. J Med Virol 82(9), 1527-1536.

28 Puche, M.L., Kay-Valero, S., Michelli, P., Oropeza, M.D., Loureiro, C.L., Devesa, M., Dagher, L., Pujol, F.H., 2016. Genetic diversity of hepatitis B virus and mutations associated to hepatocellular carcinoma in patients from Venezuela, with different stages of liver disease. Invest Clin 57(1), 38-46.

29 Shibayama, T., Masuda, G., Ajisawa, A., Hiruma, K., Tsuda, F., Nishizawa, T., Takahashi, M., Okamoto, H., 2005. Characterization of seven genotypes (A to E, G and H) of hepatitis B virus recovered from Japanese patients infected with human immunodeficiency virus type 1. J Med Virol 76(1), 24-32.

30 Sunbul, M., Sugiyama, M., Kurbanov, F., Leblebicioglu, H., Khan, A., Elkady, A., Tanaka, Y., Mizokami, M., 2013. Specific mutations of basal core promoter are associated with chronic liver disease in hepatitis B virus subgenotype D1 prevalent in Turkey. Microbiol Immunol 57(2), 122-129.

31 Tatsukawa, M., Takaki, A., Shiraha, H., Koike, K., Iwasaki, Y., Kobashi, H., Fujioka, S., Sakaguchi, K., Yamamoto, K., 2011. Hepatitis B virus core promoter mutations G1613A and C1653T are significantly associated with hepatocellular carcinoma in genotype C HBV-infected patients. BMC Cancer 11, 458.

32 Martinez, A.A., Zaldivar, Y., Hong, C., Alvarado-Mora, M.V., Smith, R., Ortiz, A.Y., Pinho, J.R., Cristina, J., Pascale, J.M., 2013. Molecular characterisation of hepatitis B virus in the resident Chinese population in Panama City. Mem Inst Oswaldo Cruz 108(5), 541-547.

33 Pezzano, S.C., Torres, C., Fainboim, H.A., Bouzas, M.B., Schroder, T., Giuliano, S.F., Paz, S., Alvarez, E., Campos, R.H., Mbayed, V.A., 2011. Hepatitis B virus in Buenos Aires, Argentina: genotypes, virological characteristics and clinical outcomes. Clin Microbiol Infect 17(2), 223-231.

34 Luo, K., Liu, Z., He, H., Peng, J., Liang, W., Dai, W., Hou, J., 2004. The putative recombination of hepatitis B virus genotype B with pre-C/C region of genotype C. Virus Genes 29(1), 31-41.

35 Skelton, M., Kew, M.C., Kramvis, A., 2012. Distinct mutant hepatitis B virus genomes, with alterations in all four open reading frames, in a single South African hepatocellular carcinoma patient. Virus Res 163(1), 59-65.
