# Supplementary material for: Characterization of hepatitis B virus with complex structural variations
Source: BMC Microbiol. 2018 Dec 3;18:202. doi: 10.1186/s12866-018-1350-1 (PMC6276219; doi:10.1186/s12866-018-1350-1)
Supplement: Supplementary file 3 — Figure S1. Patterns of complex SVs in HBV strains. (DOCX 929 kb) [file 12866_2018_1350_MOESM3_ESM.docx]

**FIGURE S1. Patterns of complex SVs in HBV strains.**

Complex SV patterns of strains Nos. 17-19, 21-24, 26-32, 34-36, 38-58, 60-70 are shown in Figure S1A -AM, respectively. From Figure S1P-AM, reference sequences of HBV/A to HBV/E were omitted.


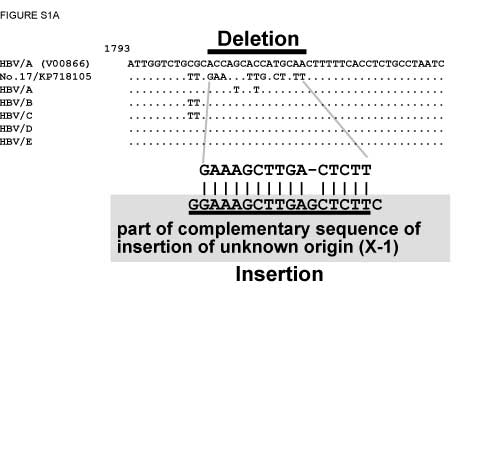


**
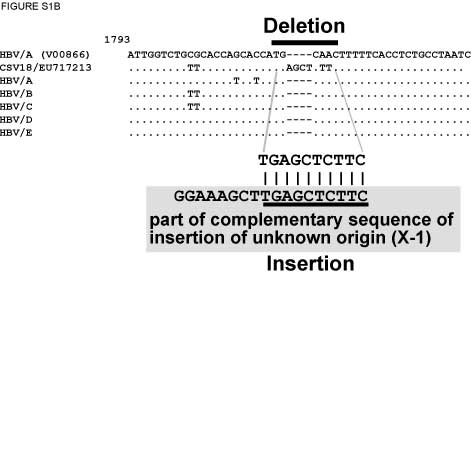
**

**
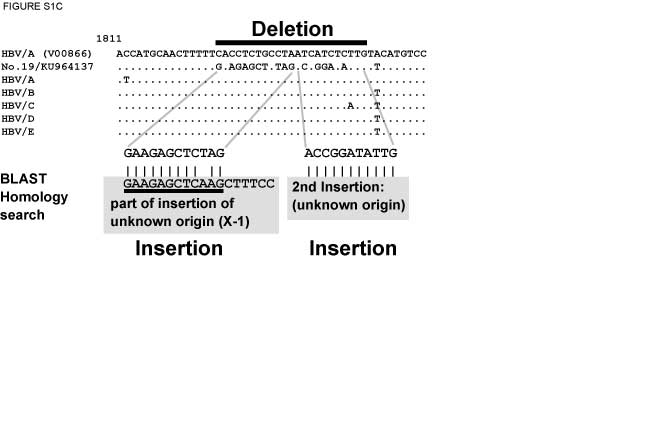
**

**
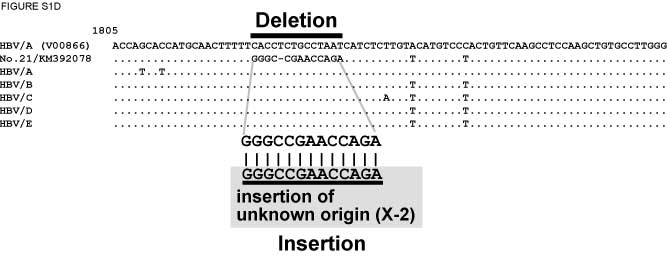
**

**
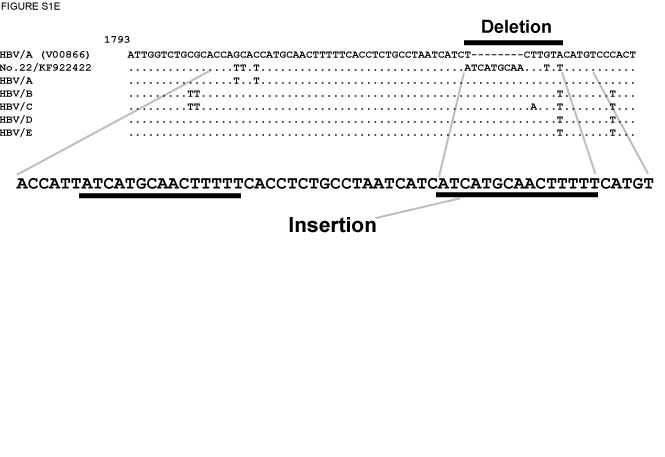
**

**
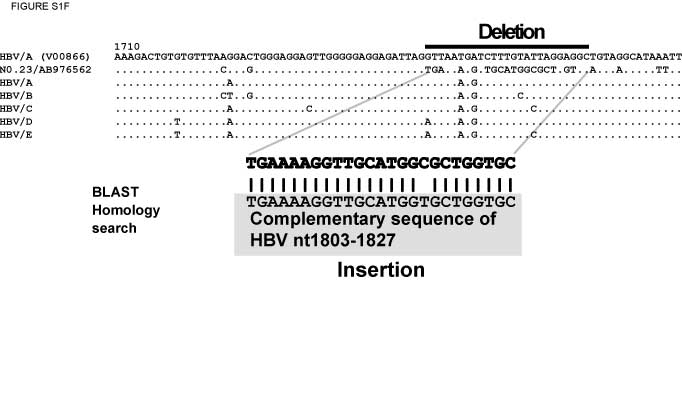
**

**
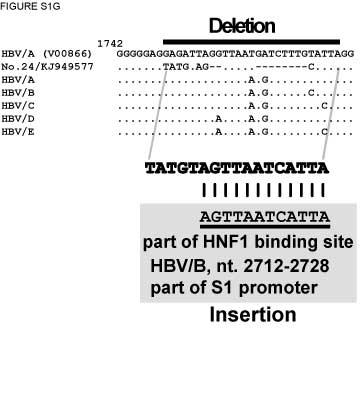
**

**
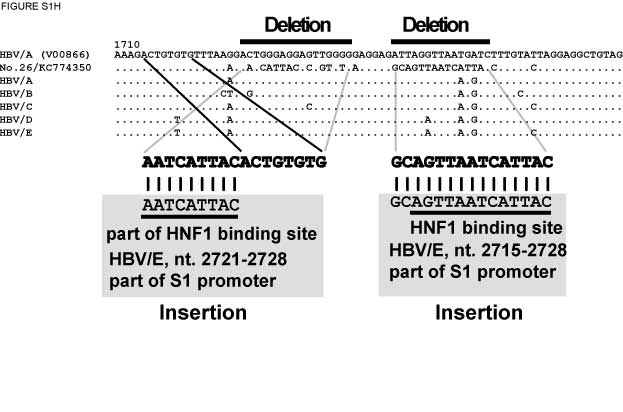
**

**
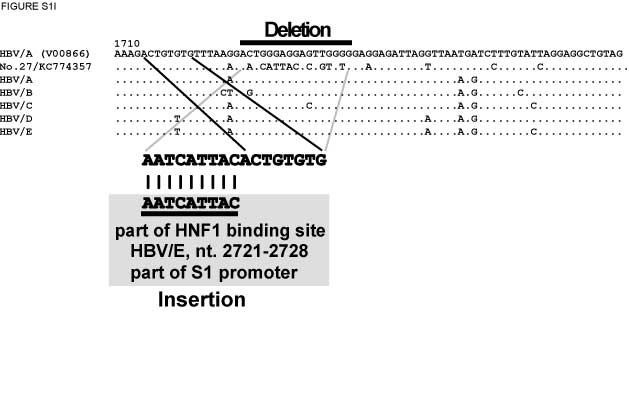
**

**
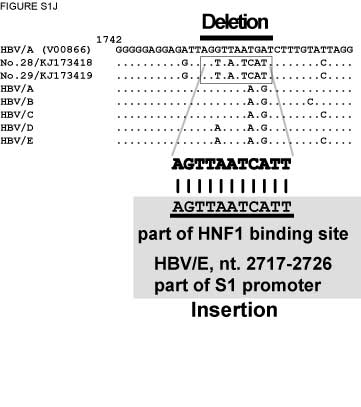
**

**
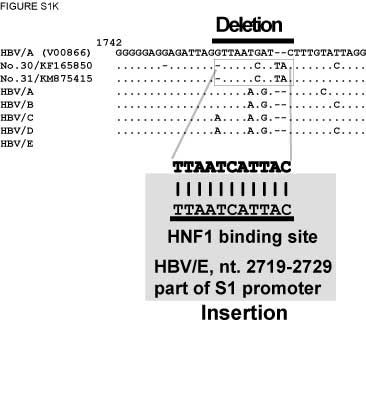
**

**
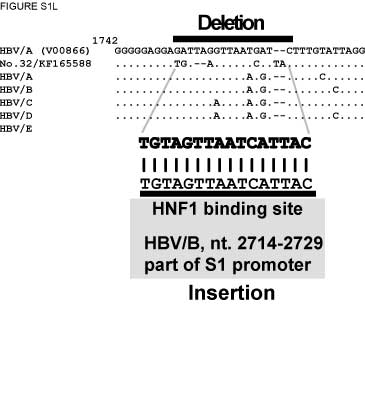
**

**
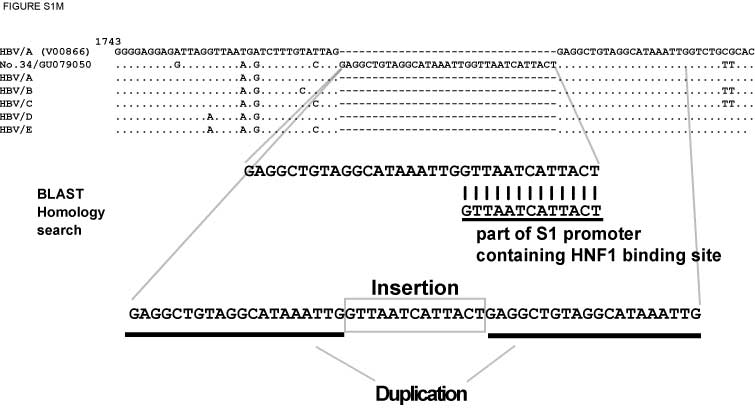
**

**
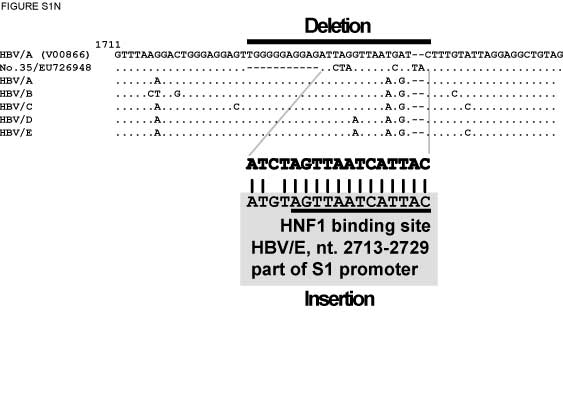
**

**
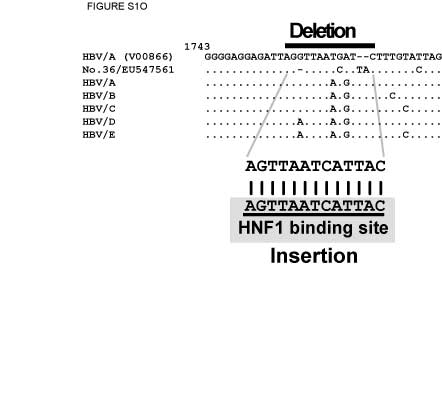
**

**
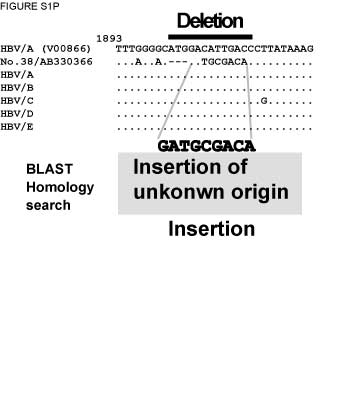
**

**
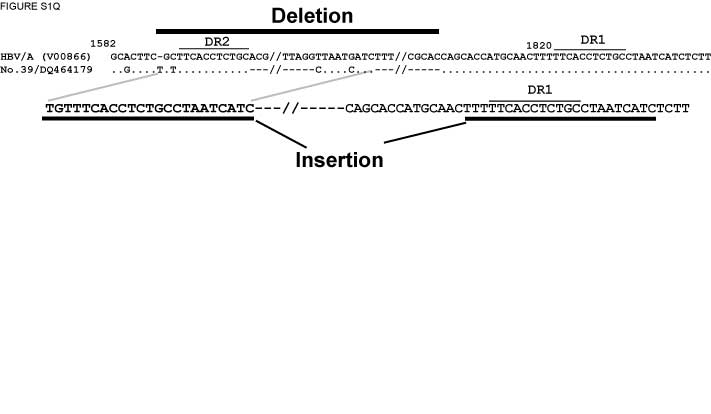
**

**
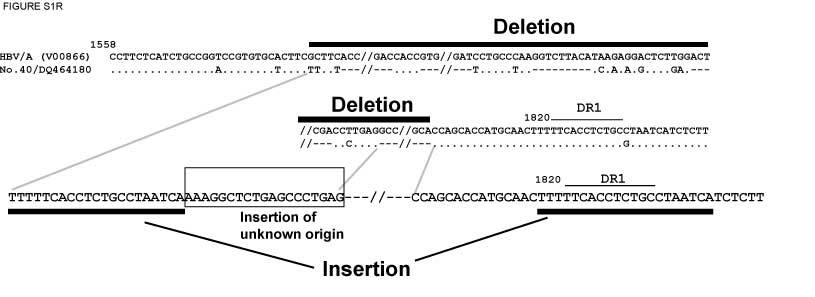
**

**
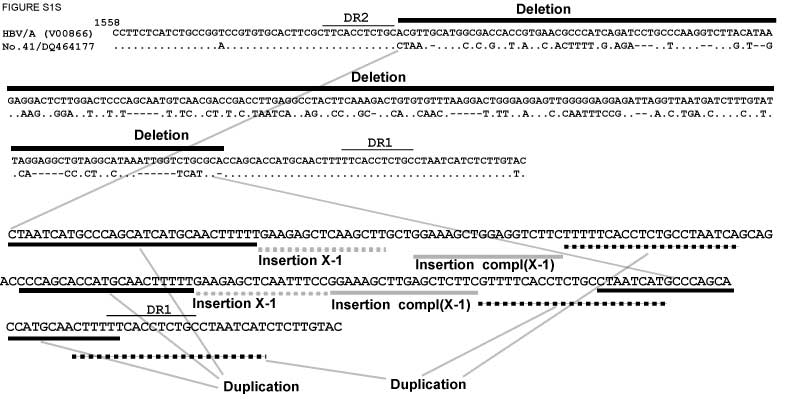
**

**
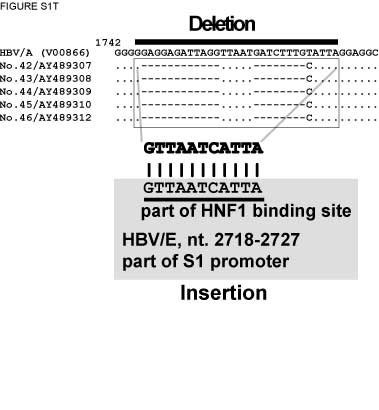
**

**
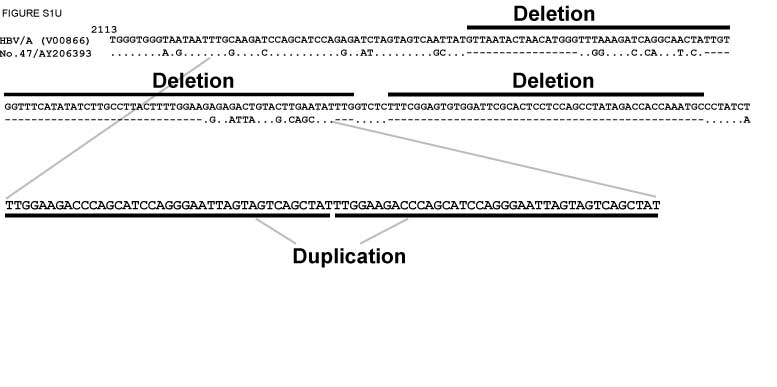
**

**
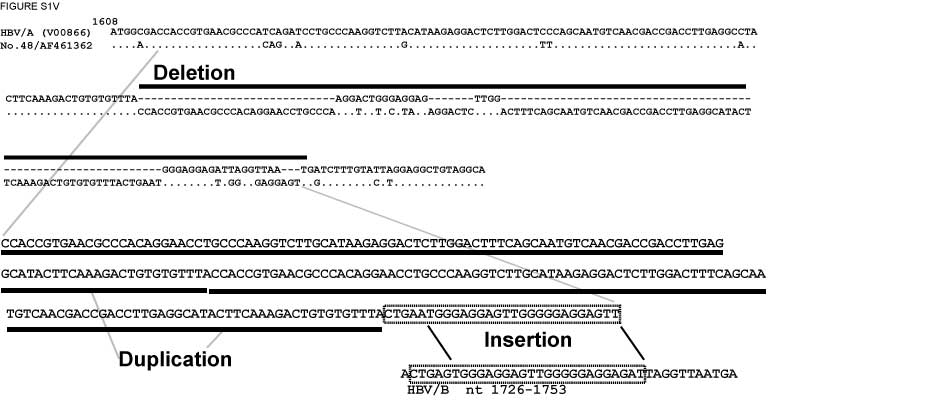
**

**
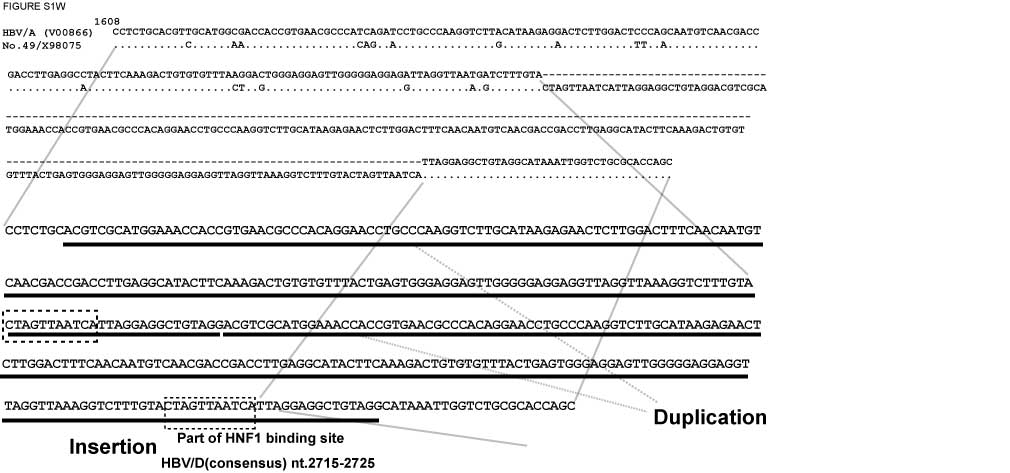
**

**
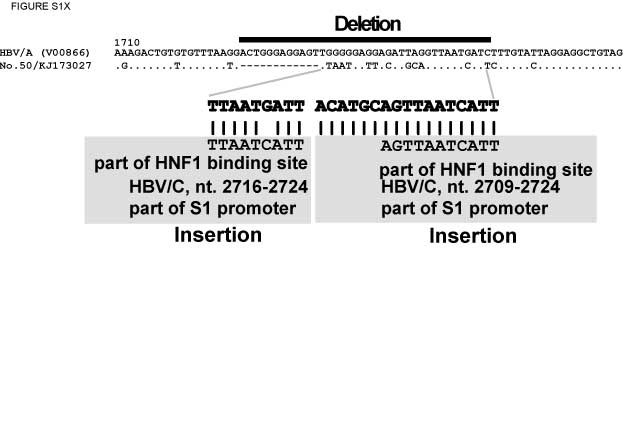
**

**
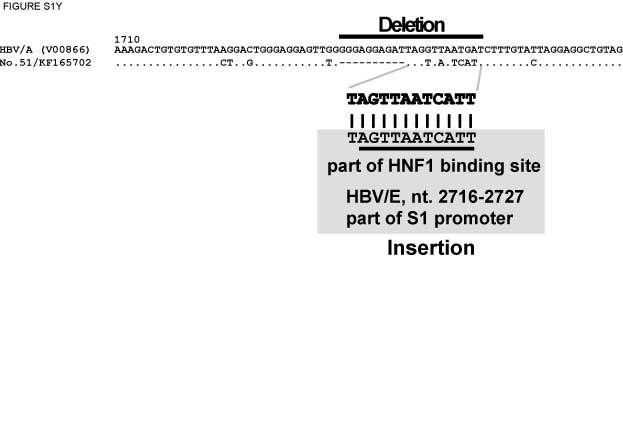
**

**
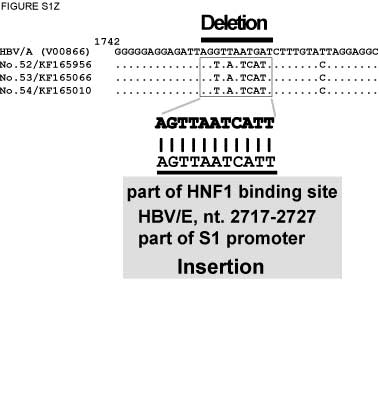
**

**
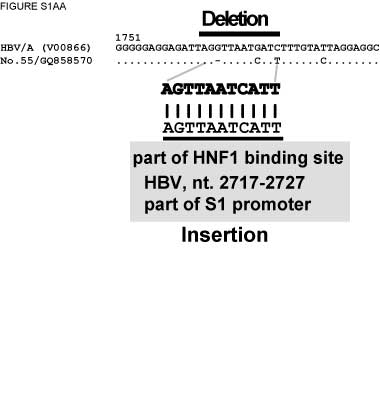
**

**
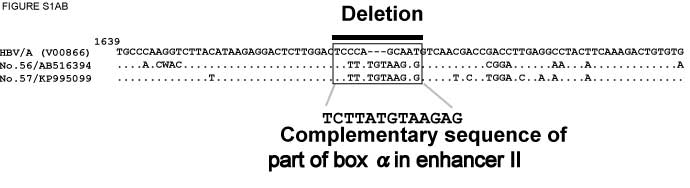
**

**
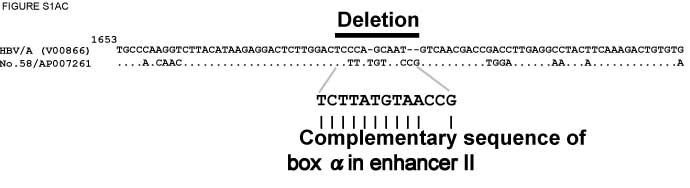
**

**
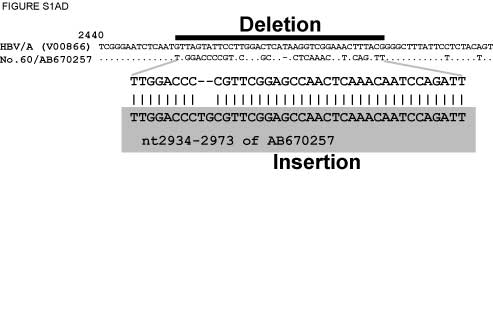
**

**
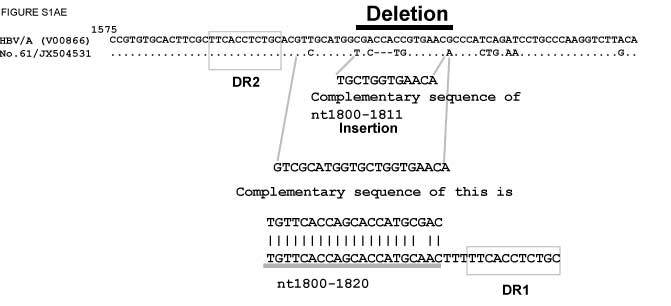
**

**
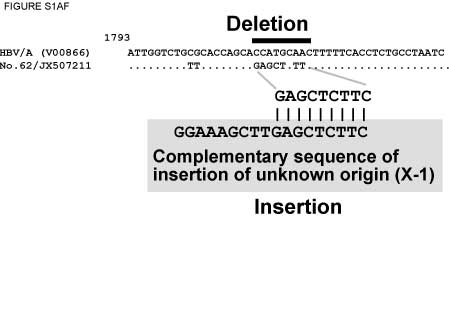
**

**
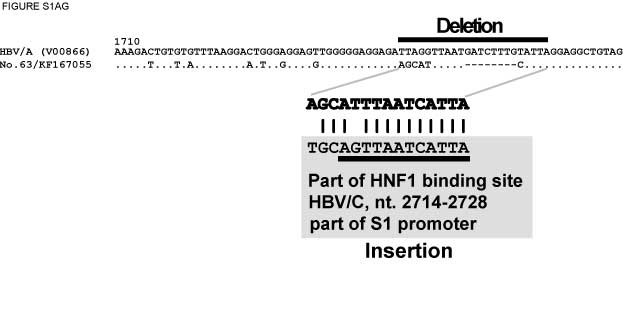
**

**
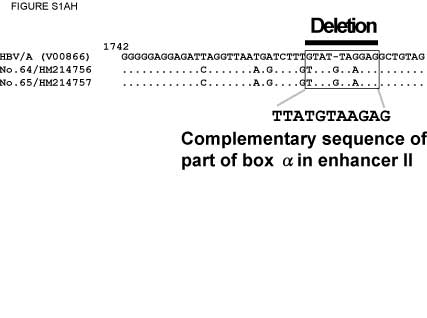
**

**
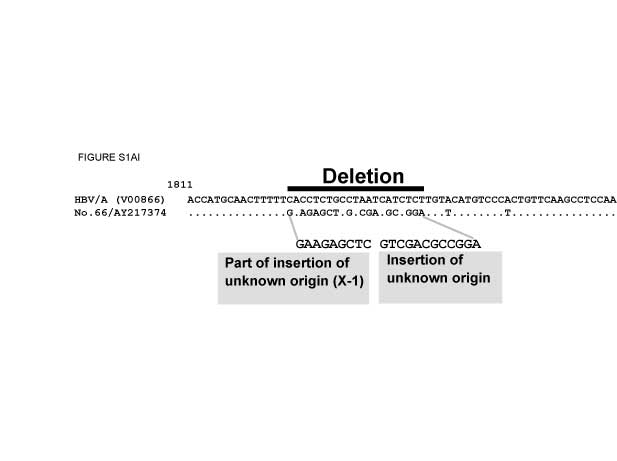
**

**
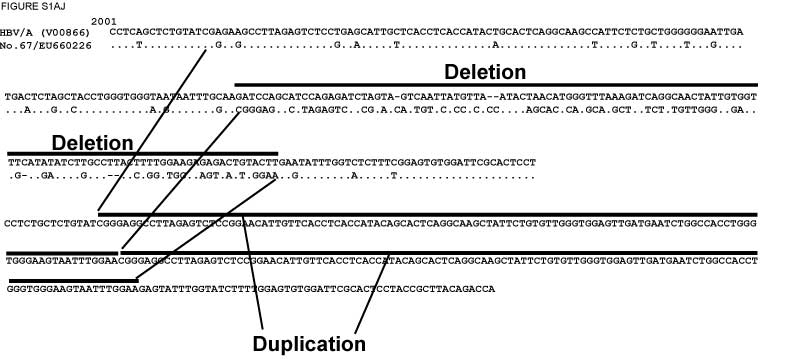
**

**
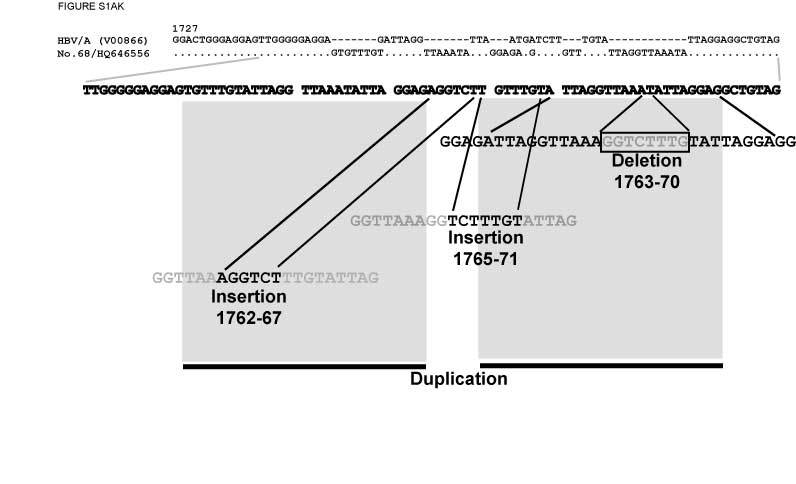
**

**
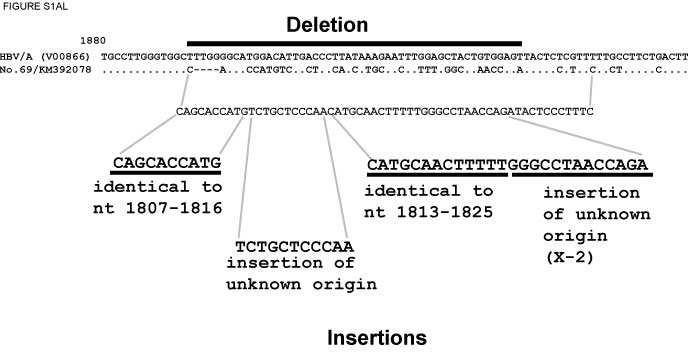
**

**
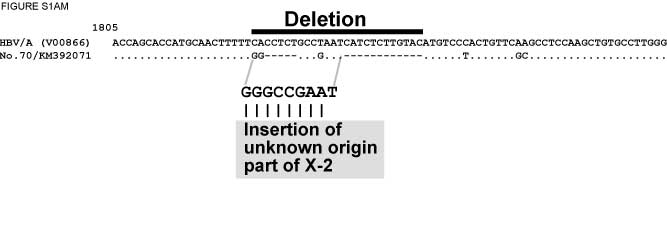
**
