# Supplementary material for: Characterization of hepatitis B virus with complex structural variations
Source: BMC Microbiol. 2018 Dec 3;18:202. doi: 10.1186/s12866-018-1350-1 (PMC6276219; doi:10.1186/s12866-018-1350-1)
Supplement: Supplementary file 4 — Figure S2. HBV genotypes. (DOCX 54 kb) [file 12866_2018_1350_MOESM4_ESM.docx]

**FIGURE S2. HBV genotypes.**

Phylogenetic analysis of HBV was performed by the neighbor-joining method for the 44 strains with complete genetic sequences available. HBV strains with complex SVs are shown in bold letters.

**
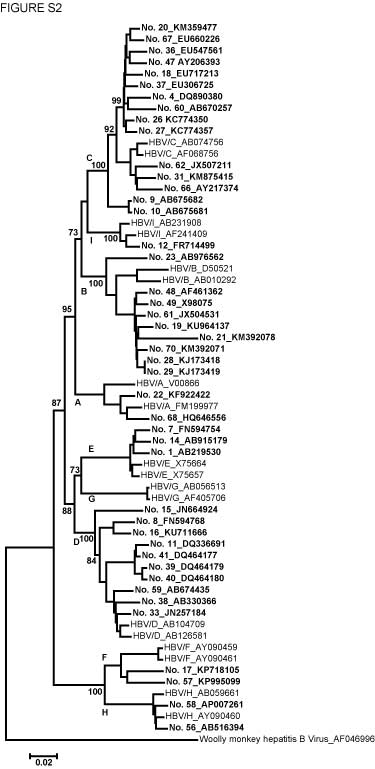
**
