# Supplementary material for: Characterization of hepatitis B virus with complex structural variations
Source: BMC Microbiol. 2018 Dec 3;18:202. doi: 10.1186/s12866-018-1350-1 (PMC6276219; doi:10.1186/s12866-018-1350-1)
Supplement: Supplementary file 5 — Figure S3. Insertional motif genetic sequences. (DOCX 93 kb) [file 12866_2018_1350_MOESM5_ESM.docx]

**FIGURE S3. Insertional motif genetic sequences.** Numbers show the HBV strain number in Additional file 1: Table S1. (A) Insertional motif genetic sequence of HNF1 binding site in pre-S1 promoter region is shown. The reference sequence of V00866 (HBV/A) was aligned with consensus genetic sequences of HBV/A to HBV/E. Of more than 30 HNF1 binding site insertional genetic sequences, 24 insertions are shown. (B) Insertions of unknown origin (X-1) are shown. In X-1, genetic sequences complementary to X-1 were also observed as insertional motif. (C) Insertions of unknown origin (X-2) are shown. (D) Insertions of a sequence complementary to part of box α in enhancer II are shown. In the initial search, this genetic sequence was identified as insertion of unknown origin (X-3), and during this analysis, it was clarified that X-3 is complementary to part of box α in enhancer II.

**
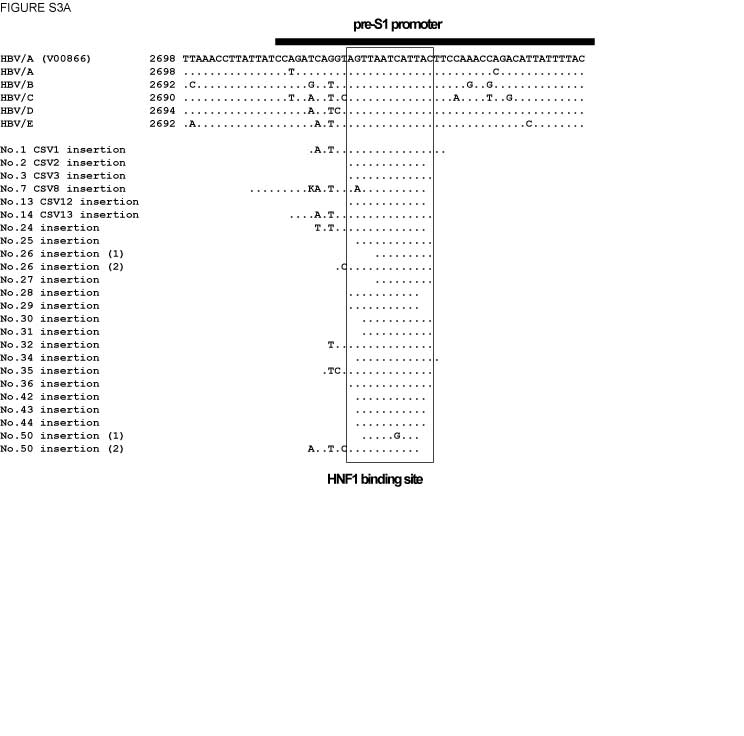
**

**
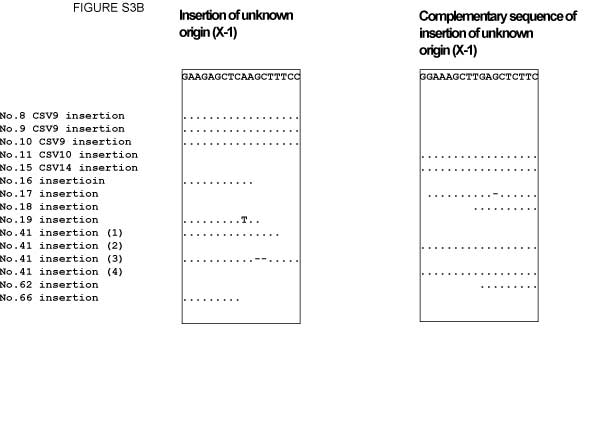
**

**
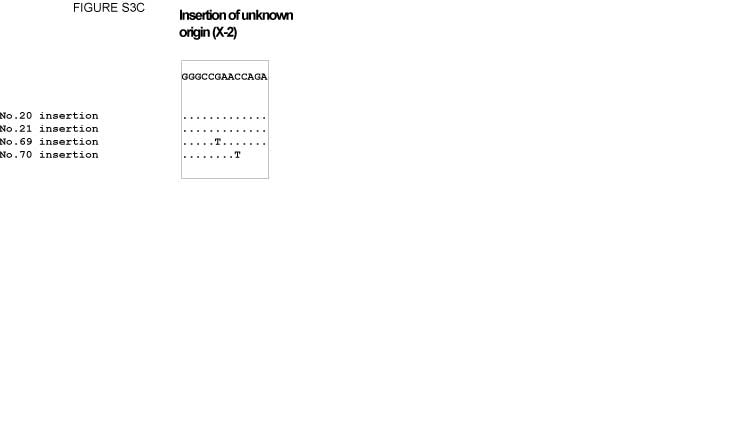
**

**
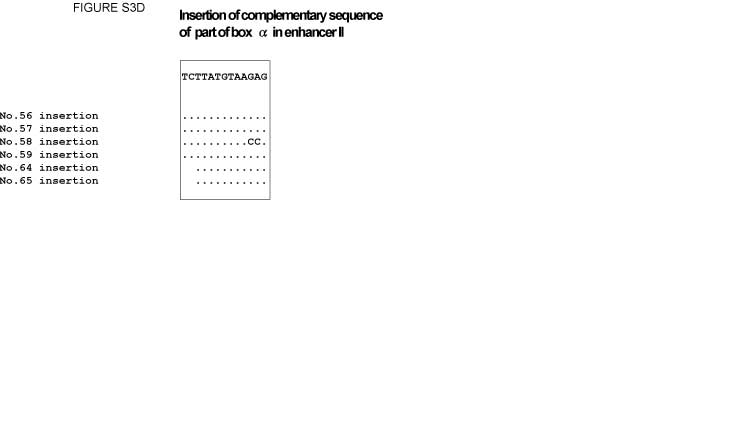
**
